# Households’ Direct Economic Burden Associated with Chronic Non-Communicable Diseases in Saudi Arabia

**DOI:** 10.3390/ijerph19159736

**Published:** 2022-08-08

**Authors:** Ziyad S. Almalki, Abdullah K. Alahmari, Nasser Alqahtani, Abdulaziz Ibrahim Alzarea, Ahmed M. Alshehri, Abdulrahman M. Alruwaybiah, Bader A. Alanazi, Abdulhadi M. Alqahtani, Nehad J. Ahmed

**Affiliations:** 1 Department of Clinical Pharmacy, College of Pharmacy, Prince Sattam Bin Abdulaziz University, Riyadh 16278, Saudi Arabia; 2 Drug & Pharmaceutical Affairs, Riyadh First Health Cluster (C1) at Ministry of Health, Riyadh 12233, Saudi Arabia; 3 Department of Clinical Pharmacy, College of Pharmacy, Jouf University, Sakaka 72388, Saudi Arabia; 4 Research Center, King Fahad Medical City, Clinical Research Department, Riyadh 12231, Saudi Arabia

**Keywords:** NCDs, out-of-pocket, Saudi Arabia, healthcare burden

## Abstract

Households’ economic burden associated with chronic non-communicable diseases (NCDs) is a deterrent to healthcare access, adversely impacting patients’ health. Therefore, we investigated the extent of out-of-pocket (OOP) spending among individuals diagnosed with chronic NCDs among household members in Riyadh, Saudi Arabia. Face-to-face interviews were conducted among households in Riyadh Province from the beginning of January 2021 to the end of June 2021. The respondents were asked to record OOP spending throughout the past three months in their health. A generalized linear regression model was used to determine the effects of several factors on the level of OOP spending. A total of 39.6% of the households studied had at least one member with a chronic NCD. Diabetes patients spent an average of SAR 932 (USD 248), hypertension patients SAR 606 (USD 162), and hypothyroid patients SAR 402 (USD 107). It was shown that households with older and more educated members had greater OOP spending. Households with an employed head of household, more family members, higher SES status, health insurance coverage, and urban residency had significantly higher OOP expenditure. The burden of OOP spending for chronic NCD households remains high, with some disparities. The research offers important information for decision making to lower OOP cost among NCD households.

## 1. Introduction

In developing countries, socioeconomic growth, fast urbanization, and epidemiological change have contributed to an upward trend in non-communicable diseases (NCDs), also known as chronic diseases [1]. Saudi Arabia has one of the highest rates of non-communicable diseases globally and the highest in the Arabian Gulf. Evidence collected indicates a high prevalence of NCDs among the Saudi population [2]. Like most countries, the NCD burden is a public health issue, resulting in significant mortality and morbidity. NCDs claim around 83,100 deaths every year, accounting for 73% of all deaths in the Kingdom [3]. In addition to causing premature mortality, chronic diseases also hurt the economic well-being of individuals, households, and the community at large.

While the Ministry of Health (MoH) works relentlessly to convert the healthcare system into a revolutionary one that is higher in quality, more efficient, and meets the health requirements of people [4], the country’s Vision 2030 economic revolution would be jeopardized if NCDs rates continue to rise. Because NCDs often need a wide range of therapies and long-term care, they place an increasing financial burden on patients, families, and the healthcare system as a whole as the population ages and expands [5]. NCDs are anticipated to cost Saudi Arabia USD 18.6 billion annually, or 2.8 percent of GDP. The USD 5.5 billion in direct medical costs for treating NCDs is a tiny fraction of healthcare spending, whereas the cost of hidden expenses from lost productivity will be USD 13.1 billion [6].

Although the Kingdom has offered universal access to healthcare to Saudi citizens and expats working in the public sector for many decades [4], universal access does not imply that the threat to living standards presented by medical expenditure has been eradicated. In Saudi Arabia, out-of-pocket (OOP) healthcare expenditures account for a significant share of overall healthcare spending. According to the World Health Organization (WHO), OOP spending accounted for 14.4% of total health expenditure in Saudi Arabia in 2018. This number is likely to underestimate the actual OOP spending experienced by people living with chronic NCDs [7]. Additionally, some of the country’s public and semi-public health providers may not always satisfy patients’ needs, resulting in individuals seeking medical treatment in the private sector and paying full price [8].

OOP spending is more likely to be frequent, unexpected, and supplemental while dealing with chronic diseases such as NCDs. This might make it more difficult for individuals to obtain medical treatment, thus negatively impacting their health. In addition, patients’ behavior in seeking medical care, treatment choices, financial hardship for patients, reduced adherence to medication, and delayed diagnosis have been proven to be influenced by high amounts of OOP payments [9,10,11]. Socioeconomic and other factors of families have been found to impact OOP spending [11,12,13].

Several studies have shown the influence of NCDs on OOP health spending in different countries. For example, households with NCD patients in Vietnam were 3.2 and 2.3 times more likely to experience catastrophic health expenses and poverty [14]. A different study has shown that the poorest CVD patients and their families are the most affected by catastrophic health expenditure in China, Tanzania, and India [15]. Another study found that families with NCDs are statistically more likely to incur catastrophic expenses in low- and middle-income countries than in non-NCD families [16]. In Tanzania, India, and China, low-income people with cardiovascular disease and stroke had the highest catastrophic expenditure rates. People with cancer in Iran and Vietnam and those with epilepsy in Nigeria reported the highest expenditures [17].

The Saudi MoH acknowledges that eliminating or reducing financial obstacles leads to greater accessibility to healthcare. However, there is limited understanding in the literature on the levels of OOP spending among households with chronic NCDs in Saudi Arabia. Decision-makers and policymakers must be aware of these conditions’ financial burden on individuals, households, and society and understand its determinants. One study merely focused on the relationship between income, insurance, and OOP spending [18]. To address this gap in the research, we examined the degree to which persons diagnosed with chronic NCDs are burdened by on OOP spending among household members in Riyadh, Saudi Arabia. This data is critical for determining the magnitude of the effect and assisting the government, healthcare sector, and other policymakers in developing new policies to alleviate the burden of OOP expenditure on families with NCDs.

## 2. Materials and Methods

### 2.1. Study Design

Between January and June 2021, a cross-sectional study design was used in the Saudi Arabian province of Riyadh. The Province of Riyadh is the second largest province by land after the Eastern Province, comprising 404,240 square kilometers. It is the second-largest population, behind only the Region of Mecca, with 3,681,927 Saudi households [19]. It consists of urban areas (those with at least 5000 people) and around a hundred scattered villages with fewer than 5000 people. Rural areas in Riyadh Province constitute around 8.5 percent of the city’s overall population [20]. The national GDP per capita was SAR 86,901 in 2019. Riyadh came in second place in terms of per capita GDP among Saudi Arabia’s 13 provinces, with a GDP per capita of SAR 121,395 [19].

### 2.2. Study Population and Sampling Methods

Families were included in the research if at least one member of the home had spent money on healthcare for one of the NCDs of interest (hypertension, diabetes, dyslipidemia, asthma hypothyroidism, and arthritis) three months before the interview. Excluded from the study were newlyweds and households with inadequate data on either the dependent or independent variables, or those living together for less than three months at the time of interview. The household was excluded if a home member was hospitalized or had a seasonal illness.

Given that chronic illnesses affect 15.9% of Saudi Arabia’s population [19] and with a 95 percent confidence level and 5 percent margin of error, we calculated the minimum feasible sample size of 205.47 households. Our research population in Riyadh was sampled using the WHO cluster sampling technique [21]. The sample size was adjusted to be 205.47 × 1.5 = 308 based on the STEPS survey guideline’s recommendation of a study design impact of 1.5 [22]. For this study, the final sample size was calculated as 308 × 100/90 = 343 households, assuming that 10% of respondents did not participate. The sample size was raised to improve our understanding of the problem. In all, 771 individuals over the age of 18 were surveyed.

The sample size was split into 60 clusters of 50 urban and 10 rural households from which we chose participants. Households were picked in proportion to the population of each district, which was drawn at random from each cluster. We listed all single-family households in the selected district. Each identified household was then visited by interviewers, who invited the head or main household member to participate in this study. If he or she refused, we contacted the nearest, same-type households (apartment, villa, etc.). To guarantee a broad and representative sample, just one family was included in each apartment building or complex. A backup household was employed if the primary family was unwilling or unable to engage in the interview due to the location’s remoteness or lack of road access.

### 2.3. Data Collection

Face-to-face interviews with the heads of chosen households or adults in the households who were 18 years of age or older were used to compile data on household characteristics. The interview includes questions about household members who have been diagnosed with chronic NCDs. Members of the study were asked whether they had been diagnosed in the past with any of the following chronic diseases by their doctor: hypothyroidism, arthritis, diabetes, asthma, dyslipidemia, and hypertension.

Interviews were conducted by teams consisting of twenty-one graduate students from Prince Sattam Bin Abdulaziz University’s College of Pharmacy, who were trained between 20 November and 23 November 2020, to guarantee reliability and consistency of the results. The principal investigator, who had experience in conducting qualitative research in the same areas, conducted the training and conducted structured and semi-structured interviews. Interviewers received training on research design, data collection, and interviewing techniques. It was decided to engage the assistance of three professors in training in order to act as supervisors and perform quality checks on the questionnaires. Two men and one woman were on each team. In patriarchal cultures such as Saudi Arabia, it is common that men head most households. For cultural reasons, men were interviewed by men and women by women, since in Saudi Arabia, interviewing a different gender is unusual and may be considered inappropriate. When we approached female-headed homes for interviews when males were absent because of death, divorce, or widowhood, we indicated that a female interviewer was outside the residence and sought permission for the interview to be conducted by the female interviewer. All interviews took place in a public and convenient area in the house.

### 2.4. Measures and Questionnaire

To achieve the goals of this research, the study’s investigators designed and developed a questionnaire. We utilized the Andersen Behavioral Model [23] to identify characteristics influencing OOP spending as a starting point. Even though Andersen’s principles were used, further literature checks were carried out to ensure the appropriateness of the questionnaire.

The questionnaire included OOP spending, predisposing, enabling, and need-based factors. We inquired about the OOP direct medical costs of each NCD. The 65-question questionnaire took less than 30 min to complete during the interview. Most questions were single or multiple choice, while age, OOP spending, and the total number of family members required numerical answers. Experts reviewed the study materials thoroughly to guarantee that the structured questionnaire was appropriate and relevant. The questionnaire was evaluated on 30 individuals twice, two weeks apart, as part of a pilot study to determine its reliability. Following the pilot study, no adjustments were made to the project’s questionnaire. Each completed questionnaire was examined for internal validity.

### 2.5. The Dependent Variable

The primary goal of this study was to determine the extent of OOP spending among individuals diagnosed with chronic NCDs. The International Classification defines OOP spending for health accounts as payments made at the time of using any healthcare item or service provided by any type of provider, both formal and informal, including deductibles, copayments, and coinsurance, and excluding pre-payments made in the form of insurance and any compensation received from a third party [24].

The individuals were asked to report the OOP spending incurred by the individual with NCD in the three-month period prior to receiving healthcare. The question was a continuous variable. We divided the whole OOP spending into three main groups—namely, medical services, medicines, and other expenses—to identify which categories are key drivers of increased spending. The interviews did not include questions on inpatient and outpatient use. Other expenses included informal care, hearing aids, therapeutic appliances, and equipment. Spending on nutritional supplements and alternative and traditional medicine was also included in OOP spending. All results were divided by three to estimate the average monthly OOP spending at the household level.

### 2.6. Independent Variables

Predisposing factors include information related to an individual with NCD, such as gender, age, marital status (not married or married), living condition (Alone, With family), and educational status (illiterate/read/write, school degree, or higher education) and information related to the household, such as the total number of family members in the household (continuous variable) and the presence of at least one member less than 14 years old in the household (yes or no).

Enabling factors to include household head employment status (unemployed or employed), residential area (rural or urban), health insurance (yes or no), and having a regular doctor (yes or no). Taking into consideration the high level of unreliability [25], including the reluctance of individuals to reveal accurate information about their income [26], researchers consider a valid country-specific socioeconomic status index (SES index) as a better economic indicator for the household than income. Our study measured SES status using the continuous Saudi-based SES index, where information from the households’ asset holdings was used [27]. The Household SES index was ranked into five quintiles. The quintile included the poorest households labeled as the first quintile and the quintile containing the wealthiest households labeled as the fifth quintile.

Need-based characteristics include level of physical activity of individuals with NCDs (active: at least 75 min of vigorous activity or at least 150 min of moderate or vigorous activity per week; moderately active: 1 to 74 min of vigorous activity or 1 to 149 min of moderate or vigorous activity per week; inactive: 0 min of moderate or vigorous activity per week), presence of at least one member with a disability in the household (yes or no), and self-reported health status of individuals with NCDs (very good, good, average, poor, and very poor).

### 2.7. Ethics, Consent, and Permissions

This study was subjected to an MoH institutional review board evaluation and approved (IRB#00010471). The study met ethical standards in agreement with the World Medical Association Declaration of Helsinki. The data anonymization and aggregation were used to ensure the confidentiality of the information. Before carrying out the research, prior written informed consent was acquired from each respondent, and no incentives were offered for participation.

### 2.8. Statistical Analysis

During the descriptive analysis, several aspects were evaluated regarding the characteristics of our sampled population who reported having at least one NCD. Categorical variables were represented as numbers and percentages, and the median (interquartile range (IQR)) was utilized to characterize continuous data such as OOP spending statistics. A box and whisker plot was used to illustrate the wide variation of OOP spending across several segments, such as medical services, medicines, and other expenses involved with each NCD. Several assumptions about linear regression were examined, including multi-collinearity, independence, homoscedasticity, and normality/linearity assumption tests. The Shapiro–Wilk test was significant, and the data were positively skewed to the right such that the linear regression model’s normality assumption was not met. As a result, a generalized linear regression model (GLM) for Gamma-distributed dependent variable took into consideration the special features of our data in order to determine how predisposing, enabling, and need-based factors affected levels of OOP spending. The Gamma distribution can model monetary variables with their typical right-skewed distribution. GLM models for Gamma-distributed variables have been proven to handle non-normality and heteroscedasticity data using the Box–Cox transformation. Significant associations in the model were determined at the 5% alpha level (*p* < 0.05). All data were analyzed using SAS version 9.4 (SAS Institute Inc., Cary, NY, USA). The result was mainly presented in Saudi Arabian Riyals (SAR) and US dollars when possible, and the exchange rate of the SAR against the US Dollar is (USD 1 = SAR 3.75).

## 3. Results

**Figure 1:**
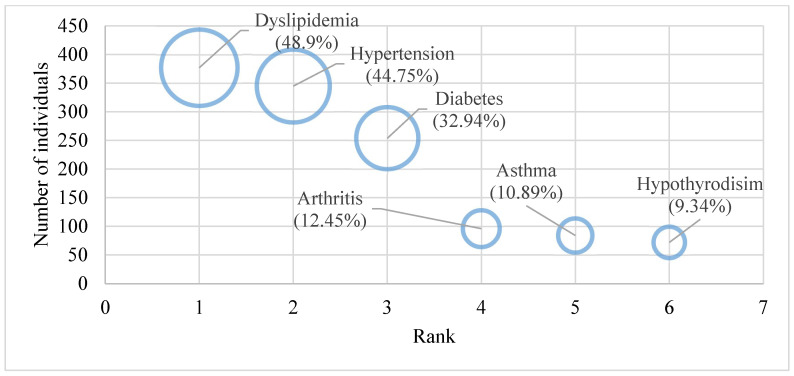
Distribution of individuals by NCDs and rank order in the sample.

Among the 1298 households visited, we excluded newlywed families (19) and families with a member diagnosed with an NCD of interest who had an acute illness (24). Sixty-two households that declined to be interviewed were replaced. This survey involved the interviewing of 1255 households. After excluding the 79 (6.3% of the total) households that could not provide all the requested information, we had 1176 households remaining. A total of 39.6 percent of the families investigated had a chronic NCD of interest in at least one of their members. In total, 771 individuals had at least one chronic NCD. Regarding the prevalence of self-reported chronic NCDs, dyslipidemia was the top-ranked NCDs, accounting for almost half of the studied population (48.9%). Hypertension was the second-highest ranked disease in the study, while diabetes was ranked the third-highest NCD in the study at 32.94% (Figure 1). 

More than three-quarters of individuals with NCDs (75.49%) were male, and the majority of individuals were aged twenty-nine years and younger (27.3%) or between thirty and thirty-nine years (27.11%). The study also revealed that the majority of individuals were married (82.1%), lived together with their families (91.83%), had a school degree (43.32%), were employed (54.6%), and were physically active (37.87%) (Table 1).

The majority of households had 4–6 members living in the household (44.36%), had at least one child up to 14 years old (52.79%), resided in urban areas (77.56%), and belonged to poor and middle SES quintiles (28.4% and 23.87%, respectively). Most individuals do not purchase health insurance (56.84%) and have a regular doctor (53.57%).

Box-and-whisker plot comparisons reported OOP spending for individuals with different NCDs: total OOP spending and OOP spending related to healthcare services, medicines, and other expenses. The boxes represent the first and third quartile range (50% of the data). The horizontal line in the box interior represents the median. The upper and lower lines represent the minimum and maximum values. Outliers are not presented. 

Median OOPHEs were highest for individuals with diabetes. On average, the median total OOP spending was SAR 932 (USD 248) (interquartile range (IQR), SAR 481–SAR 1086) for individuals with diabetes, SAR 606 (USD 162) (IQR, SAR 400–SAR 805) for hypertension, and SAR 402 (USD 107) (IQR, SAR 231–SAR 543) for hypothyroidism.

**Figure 2:**
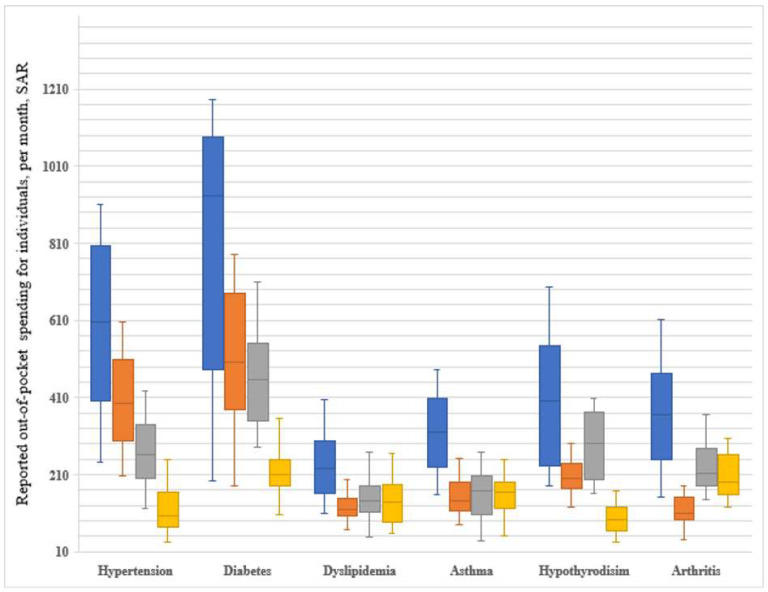
Box-and-whisker plots of OOP spending per individual with NCDs per month.

The total expenses across all individuals with different NCDs appear to be driven mainly by health services expenses, in the case of individuals affected with diabetes, hypertension, and medicine for those with hypothyroidism and arthritis. Individuals with diabetes spent the most considerable portion of OOP spending on healthcare services, with a median of SAR 501 (USD 134) (IQR, SAR 380–SAR 680) per month. Similarly, the greatest share of the OOP spending spent on medicines among individuals with hypertension was a median of SAR 396 (USD 106) (IQR, SAR 297–SAR 507); the medicine expenses drive the total OOP spending in individuals with hypothyroidism or arthritis with a median of SAR 291 (USD 78) (IQR, SAR 197–SAR 373), or a median of SAR 213 (USD 57) (IQR, SAR 181–SAR 279), respectively (Figure 2).

According to GLM results (Table 2, across almost all NCDs, we found several characteristics that significantly affect the level of OOP spending. An older, better educated, and employed household head was associated with a positive coefficient. At the household level, the increased number of family members and residence in the urban area were positively associated with OOP spending.

The findings also show that OOP spending increased significantly with the household SES increase. Furthermore, households covered by health insurance plans are remarkably associated with higher OOP spending than those without health insurance. The findings found that having a regular doctor has a negative effect on the level of OOP spending. Finally, OOP spending was higher among inactive individuals than among active individuals. Additionally, the total OOP expenses were higher among individuals with poorer general health than those with very good health.

## 4. Discussion

Although progress has been made in several nations, overall OOP expenditure impedes universal health care and financial security, particularly for people suffering from chronic NCDs. Determining the correct amount of OOP spending and identifying sociodemographic categories that may be disproportionately impacted are essential steps in the process. As a result, the major focus of this research is on the burden of OOP spending put on Saudi Arabian families as a result of NCDs.

We found that the amount of OOP money households had to spend on health care for individuals with hypertension or diabetes was the highest compared to other NCDs, mirrored in the international literature [28,29,30]. According to WHO, ‘Big Four’ NCDs, two of the four NCDs, hypertension and diabetes, have a significant financial impact on people in terms of direct costs. For policymakers and health care providers, these results are especially useful in understanding the financial elements of the increasing burden of NCDs, especially hypertension and diabetes.

As a result, our research sheds insight on the components of health care that lead to increased out-of-pocket costs. An individual suffering from hypertension or diabetes has higher OOP spending for services and drugs, as shown by examining the breakdown of OOP spending. These results are congruent with those of other recent investigations. Shumet et al. observed that the most prevalent causes of catastrophic health spending among diabetic patients were high-cost services and medicines [31]. Other findings have shown that chronic disease-affected families are much more likely to spend on medicines than matched control unaffected families [32]. These findings are predictable, given the complex care and treatment needs of those patients [33]. 

Additionally, the study examined the relationship between predisposing, enabling, and need-based variables and the magnitude of OOP spending among patients with NCDs. Our data indicated that the risk of OOP spending was greater among older adults diagnosed with hypertension, diabetes, and dyslipidemia, which is in line with previous results [28]. This increased likelihood is due to the older generation’s greater demand for and utilization of healthcare services than younger age groups. Our research also found that a higher educational level was associated with higher OOP spending, probably due to higher awareness of the importance of health and more knowledge about healthcare alternatives. This conclusion corroborates research performed in other nations [34,35]. Our study also discovered that employment status is a major factor. Our finding indicated that employed heads of households are more likely to have larger OOP spending than those who are unemployed. This conclusion is consistent with findings from other nations’ investigations [36,37].

Unsurprisingly, the present study’s findings indicated that the variable “number of family members” had a noticeable impact on OOP spending. The results also agree with studies from different countries such as China and Serbia [38,39]. Another unexpected finding is that the level of OOP spending was much greater in urban households than in rural households. This appears counterintuitive and contradictory to what is observed in other countries [28,40,41]. Urban regions may be more likely to have superior medical facilities and specialists, and patients with chronic NCDs tend to live nearby.

Amongst the enabling component of households belonging to the household’s SES, our results imply that the wealthier households are more likely to spend more OOP than poor households. It was fair to assume that the lower class had a limited ability to obtain medical treatment and a tendency to avoid physicians, due to financial difficulties, when encountering chronic illness [42]. This finding emphasized the vulnerable position of the poor population when seeking health services. 

Although data from various countries indicates that insured households incur lower OOP spending [34,43], our results show that households covered with health insurance spend more on OOP spending. A critical observation on the association perhaps indicates that the health insurance is not financially protective enough to keep OOP spending under control. However, denying the value of health insurance solely based on this observation might be deceptive. The high level of OOP spending might be partially explained by improved access to care and increased healthcare services utilization by insured families. On the other hand, it could be attributed to adverse selection; that is, families make insurance purchase decisions based on their estimate of risks; as such, families who have chronic NCDs are often more likely to buy insurance coverage and use more healthcare services. Furthermore, insured households with generous plans have incentives to overutilize services, referred to in the economic literature as moral hazards [44].

In addition, having a regular doctor has a strong detrimental influence on the OOP spending level. Usually, households with a doctor who visits them regularly have better access to preventive services and are more likely to follow the doctor’s prescriptions. Consequently, such patients are less likely than others to return for follow-up appointments after an emergency department visit, and they have lower rates of health and drug complications [45,46].

Our finding indicates that being physically inactive significantly increases the level of OOPE among NCDs patients. Physical activity may be an effective policy tool for reducing the economic burden associated with out-of-pocket health care costs [47]. Finally, health status was a critical component in determining a person’s tendency for high OOP levels. People’s OOP steadily rises as their health worsens, supported by the literature [48].

There are a few limitations in the survey data and the methodology to consider. First, the current data was based on a cross-sectional survey, where self-reported OOP spending was used, which is also common to most previous expenditure studies. Thus, the design may be affected by recall bias and reporting bias and is not verified by other sources. Future studies may address these problems by using administrative health data. Second, the study population consisted of households that had received care in the three months preceding the interview; therefore, OOP spending may be overestimated compared to the general population. Third, in most nations, the pandemic lowered the number of individuals seeking medical care [49], but our data were inadequate to determine if there were substantial changes in OOP expenditures due to the pandemic. Finally, some households are not easily accessible within the sample area due to poor road infrastructure and thus cannot be sampled. In a way, these could have occasioned some element of technical bias because other households were chosen to take the place of those unavailable for interviews.

Despite these limitations, the evidence generated by our study has important policy implications in Saudi Arabia. In Saudi Arabia, as part of the Health Sector Transformation Program, numerous financial reforms have been implemented to address people’s health needs. One of these reforms is a Program for Health Assurance and Purchasing (PHAP) to be national single-payer health insurance to ensure free and accessible care is available to all citizens and legal residents through the newly MOH-corporatized providers and other governmental providers. However, it must be carefully designed before implementation, and stimulating financial risk protection strategies through lowering OOP spending should be prioritized. Vulnerable groups, such as people with NCDs, would have an exception to the cost of some services and medications, including lower copayments and subsidization of vital drugs.

Another reform is a system of supplementary health insurance (SHI) which will allow most citizens and residents to add additional benefits [50]. These reforms are expected to reduce OOP spending and provide financial protection against high OOP spending only if policymakers consider the impact of these policies on persons with chronic NCDs and their families. However, their effectiveness can be assessed to improve access to healthcare and reduce OOP spending by the families. 

From the clinical practice perspective, it is critical to maximize efficiency for service delivery to patients with chronic NCDs such as hypertension and diabetes by adopting the patient-centered medical home (PCMH) model of care. This model is based on the same principles as the Chronic Care Model, with the primary goal of providing patients with organized, proactive, and coordinated care rather than episodic treatments to improve outcomes while also lowering management costs [51,52]. 

## 5. Conclusions

According to the results of this study, households spend a significant amount of money to take care of family members diagnosed with chronic NCDs, most notably hypertension and diabetes. Most of these out-of-pocket expenses may be attributed to the cost of services and treatment. Studying the factors that determine out-of-pocket expenditure allowed researchers to find information that might help reduce OOP costs for households with chronic NCDs. These findings may provide information that policymakers may use to their advantage when developing policies to execute future healthcare reform programs. Saudi Arabia’s national health insurance program must be appropriately designed to minimize out-of-pocket expenditures among those who have NCDs before it can be put into effect.

## Figures and Tables

**Table 1:** The surveyed individuals with NCD’ characteristics (N = 771).

Characteristics	N	Percentage (%)
**Predisposing**		
** Gender**		
Female	189	24.51
Male	582	75.49
** Age group, year**		
≤29	321	27.3
30–39	209	27.11
40–49	158	20.49
50–59	160	20.75
≥60	136	17.64
** Marital status**		
Not married	138	17.9
Married	633	82.1
** Living condition**		
Alone	63	8.17
With family	708	91.83
** Educational status**		
Illiterate/read/write	116	15.05
School degree	334	43.32
Higher education	321	41.63
** Number of family members in the household**		
≤3	254	32.94
4–6	342	44.36
≥7	175	22.7
** Presence of at least one member less than 14 years in the household**		
No	364	47.21
Yes	407	52.79
**Enabling**		
** Residential area**		
Rural	173	22.44
Urban	598	77.56
**Household head employment status**		
Unemployed	350	45.4
Employed	421	54.6
** SES index**		
0–20% Poorest	102	13.23
20–40% Poor	219	28.4
40–60% Middle	184	23.87
60–80% Wealthy	153	19.84
80–100% Most wealthy	113	14.66
** Health Insurance**		
No	648	56.84
Yes	492	43.16
** Having a regular doctor**		
No	413	53.57
Yes	358	46.43
**Need-based**		
** Level of physical activity**		
Active	292	37.87
Moderately active	272	35.28
Inactive	207	26.85
** Presence of at least one member with a disability**		
No	563	75.37
Yes	184	24.63
**NCDs**		
Hypertension	345	44.75
Diabetes	254	32.94
Asthma	84	10.89
Dyslipidemia	377	48.9
Hypothyroidism	72	9.34
Arthritis	96	12.45
**Health in the last two months before the interview**		
Very Poor	117	15.18
Poor	178	23.09
Average	168	21.79
Good	119	15.43
Very Good	189	24.51

Abbreviations: SES: socioeconomic status.

**Table 2:** Predictors of the total OOP spending level due to NCDs: generalized linear model.

Independent Variable	Hypertension	Diabetes	Dyslipidemia	Asthma	Hypothyroidism	Arthritis
	Adjusted β-Coefficient (SE)					
**Gender (reference category: Female)**						
Male	0.209 (0.073) *	0.099 (0.101)	0.385 (0.075) *	−0.371 (0.143) *	−0.0786 (0.232)	0.481 (0.192) *
**Age group, year (reference category: ≤29)**						
30–39	−0.166 (0.098)	0.045 (0.142)	0.149 (0.211)	−0.356 (0.224)	0.368 (0.339)	0.313 (0.327)
40–49	0.091 (0.103)	0.106 (0.126)	0.199 (0.107) *	−0.071 (0.213)	0.156 (0.317)	0.001 (0.274)
50–59	0.095 (0.090) *	0.311 (0.121) *	0.072 (0.101)	0.114 (0.203)	0.035 (0.277)	0.193 (0.056) *
≥60	0.088 (0.056) **	0.457 (0.144) ***	0.113 (0.103) *	0.159 (0.337)	−0.041 (0.331)	0.478 (0.312) *
**Marital Status (reference category: Not married)**						
Married	0.184 (0.086)	0.252 (0.117)	0.187 (0.093)	0.143 (0.122)	−0.315 (0.323)	−0.087 (0.224)
**Living condition (reference category: Alone)**						
With family	0.258 (0.305)	0.011 (0.124)	0.196 (0.211)	0.093 (0.101)	0.147 (0.251)	0.109 (0.293)
**Educational status (reference category: Illiterate/read/write)**						
School degree	0.256 (0.085) *	0.267 (0.118) *	0.122 (0.094) *	0.014 (0.165)	0.114 (0.281)	0.108 (0.226)
Higher education	0.278 (0.096) ***	0.707 (0.126) **	0.588 (0.095) ***	0.427 (0.178) **	0.257 (0.295)	0.177 (0.236)
**Number of family members in the household (reference category: ≤3)**						
4–6	0.055 (0.110)	0.251 (0.128) *	0.337 (0.101) *	0.384 (0.227) *	0.297 (0.336)	0.375 (0.226) *
≥7	0.215 (0.115) **	0.431 (0.131) ***	0.494 (0.106) **	0.089 (0.241)	0.299 (0.364)	0.128 (0.244)
**At least one member less than 14 years in the household (reference category: No)**						
Yes	0.222 (0.064) ***	0.197 (0.094) **	0.253 (0.371)	0.406 (0.131) *	0.056 (0.241)	0.147 (0.183)
**Residential area (reference category: Rural)**						
Urban	0.301 (0.061) ***	0.193 (0.082) **	0.147 (0.067) **	0.046 (0.123)	0.387 (0.221) *	0.254 (0.166) *
**Household head employment status (reference category: Unemployed)**						
Employed	0.116 (0.085) **	0.171 (0.114) *	0.052 (0.087)	−0.147 (0.154)	0.211 (0.284)	−0.026 (0.212)
**The household SES index (reference category: Q1 (Poorest) (lowest 20%)**						
Q2 (Poor)	−0.023 (0.084)	0.115 (0.108) *	−0.041 (0.095)	−0.151 (0.175)	0.388 (0.381) *	0.203 (0.234)
Q3 (Middle)	0.184 (0.099) *	0.174 (0.138) *	0.079 (0.107)	0.236 (0.211) *	0.206 (0.378)	0.124 (0.296)
Q4 (Wealthy)	0.099 (0.197)	0.127 (0.112) **	0.164 (0.105) *	0.444 (0.201) **	0.591 (0.315) **	0.502 (0.274) **
Q5 (Wealthiest) (higher 20%)	0.198 (0.106) **	0.244 (0.156) **	0.193 (0.117) **	0.237 (0.212) *	0.395 (0.455)	0.364 (0.289) **
**Health Insurance (reference category: No)**						
Yes	0.201 (0.061) ***	0.141 (0.091) **	0.066 (0.166)	−0.102 (0.128)	0.114 (0.115)	0.128 (0.137) *
**Having a regular doctor (reference category: No)**						
Yes	−0.194 (0.074) **	−0.117 (0.112) *	−0.178 (0.081) **	−0.221 (0.163) *	−0.041 (0.156)	−0.058 (0.081) *
**Level of physical activity (reference category: Active)**						
Moderately active	−0.019 (0.076)	0.024 (0.111)	0.111 (0.087)	0.261 (0.174) *	0.438 (0.291) *	−0.181 (0.202)
Inactive	0.094 (0.085) *	0.222 (0.127) *	0.171 (0.093) *	0.062 (0.189)	0.309 (0.323)	0.343 (0.221) *
**At least one member with a disability in the household (reference category: No)**						
Yes	0.148 (0.163)	0.156 (0.141)	0.081 (0.071)	0.152 (0.134)	0.234 (0.224)	0.133 (0.141)
**Health in the last two months before the interview (reference category: very good)**						
Very Poor	0.218 (0.088) ***	0.233 (0.139) **	0.231 (0.097) ***	0.244 (0.041) ***	0.476 (0.301) *	0.101 (0.056) ***
Poor	0.199 (0.086) **	0.291 (0.138) *	0.332 (0.099) ***	0.246 (0.231)	0.338 (0.116) **	0.545 (0.205) ***
Average	0.041 (0.096)	0.089 (0.131)	0.237 (0.101)	0.079 (0.243)	0.389 (0.325) *	0.429 (0.205) *
Good	0.124 (0.183)	0.102 (0.129)	0.141 (0.197)	−0.155 (0.216)	0.378 (0.545)	0.511 (0.247) *

Abbreviations: SES: socioeconomic status. * *p*-value < 0.05 ** *p*-value < 0.01 *** *p*-value < 0.001. Results are controlled for gender, age, educational status, household SES index, health insurance, residential area, and NCDs.

## Data Availability

The data presented in this study are available on request from the corresponding author. The data are not publicly available due to privacy reasons.
